# Do sung speech and background noise affect sentence repetition in dyslexia?

**DOI:** 10.1007/s10339-026-01393-x

**Published:** 2026-09-18

**Authors:** Ariadne Loutrari, Ellie Packham, Roshni Vagadia, Cunmei Jiang, Fang Liu

**Affiliations:** 1 School of Psychology and Clinical Language Sciences, University of Reading, Earley Gate, Reading, RG6 6AL UK; 2 Music College, Shanghai Normal University, Shanghai, 200234 China

**Keywords:** Developmental dyslexia, Sentence repetition, Speech perception in noise, Sung speech

## Abstract

Developmental dyslexia (DD) is typically associated with slow and laborious reading, but there is also evidence pointing to speech-in-noise perception difficulties and a deficit in rapid stimulus processing. We looked at sentence repetition in 56 adult participants, half of whom had a diagnosis of DD, using a mixed design with one between-subjects variable (group) and three within-subjects variables (presentation, noise level, and semantic content). More specifically, we investigated whether sung targets could facilitate sentence repetition in DD compared to spoken ones, drawing on the idea that singing has a slower presentation rate. Further, in light of difficulties in repeating semantically meaningless targets in DD and the mixed results regarding speech-in-noise perception in this population, we examined the effects of varying semantic content (story-like, news-like, semantically anomalous) on performance, presenting targets in quiet and in noise. Results showed a significantly poorer performance in DD in quiet relative to controls but not in noise. Semantic content came out non-significant, and sung targets did not statistically affect performance. Considering a previously documented dyslexia-amusia comorbidity, we screened all participants for amusia, and—in line with previous research—we found that approximately 29% of the DD sample met the diagnostic criteria for amusia. However, amusia did not significantly affect performance. The study suggests that sentence repetition is impaired in DD and naturalistic singing does not seem to confer an advantage. As we did not isolate presentation rate as a variable, the results cannot establish that a reduced presentation rate itself has no positive effects.

## Introduction

Developmental dyslexia (henceforth DD) is a neurobiological learning disability affecting word recognition and spelling (Lyon et al. 2003). While difficulties with phonological processing (i.e., the ability to perceive, identify, store, and manipulate language sounds) have been considered as a key factor influencing reading ability (Lyon et al. 2003), more recent findings suggest that a multifactorial model including auditory processing, speech perception, and visual deficits could explain the heterogeneity of the condition better (Snowling and Hulme 2021). Various other theories have been propounded to explain the origin of the disorder (for a recent review see Wolf et al. 2024). Moreover, several comorbidities have been associated with DD, such as dyscalculia (Wilson et al. 2015), attention deficit hyperactivity disorder (Germanò et al. 2010), speech sound disorder (Mari et al. 2022), and congenital amusia (Couvignou et al. 2019; Couvignou and Kolinsky 2021). As DD is known to persist into adulthood (Bruck 1990; Reis et al. 2020; Wilson et al. 2015), the current study builds on previous work in both children and adults, considering outstanding questions on variables that may affect sentence repetition performance.

The literature points to poor repetition of both nonwords (Catts et al. 2005; Ehrhorn et al. 2021; Snowling 1981) and sentences (Dosi and Koutsipetsidou 2019; Lukic et al. 2025; Moll et al. 2015; Plaza et al. 2002) in DD. The latter impairment is more in evidence when target sentences are long and semantically meaningless (Lukic et al. 2025), a finding consistent with the positive effect of semantically meaningful content on sentence repetition in neurotypicals (Polišenská et al. 2015, 2021). In addition to the comparison of meaningful and meaningless content, one of the questions we ask in this study is whether different types of meaningful content (story-like versus news-like sentences) can also differentially affect repetition performance in individuals with DD.

Regarding auditory processing, a recent systematic review and meta-analysis revealed impairments in frequency, duration, and intensity discrimination as well as gap detection in DD (McWeeny and Norton 2024). Several studies cited below also converge on an association between DD and rapid acoustic analysis difficulties. A link between DD and rapid auditory processing was originally posited by Tallal (1980), who proposed a relationship between reading difficulties and the processing of non-verbal rapid auditory information. Tallal (1980) noted that impairments observed at the phonemic level may be accounted for by a deficit in responding to rapid auditory information. Since then, various studies have reported on rapid auditory processing difficulties in at least subgroups of people with DD (e.g. Casini et al. 2018; De Martino et al. 2001; Hari and Kiesilä, 1996; Laasonen et al. 2001; Reed, 1989). However, this link may not be causal (Marshall et al. 2001) and may stem from an attention deficit (Hari and Renvall 2001; but see Casini et al. 2018) or be modulated by additional variables (Lorusso et al. 2014).

A central question we consider is whether sentence repetition in DD is facilitated by sung versus spoken stimuli. Given that singing typically unfolds slower than speech (Ozaki et al. 2024), we expect that listeners with DD who have difficulties in perceiving rapidly changing information may find it easier to process sung sentences. However, it is of note that this assumption would not hold for songs with fast tempos or other complex characteristics. Previous research in neurotypical listeners has shown that listeners are more adept at recalling sung relative to spoken lyrics unless presentation rate is equalised across categories, meaning that any processing advantage can be pinned on the slower rate found in singing (Kilgour et al. 2000). Another study recently found that listeners recall and repeat sung targets better than spoken ones in noise (albeit not in quiet) (Loutrari et al. 2024). However, the advantage of sung targets on recall has not been borne out elsewhere in the literature (Racette and Peretz 2007).

Apart from rapidly unfolding acoustic information, simultaneous sound sources may pose high processing demands, as the listener needs to segregate the sources and suppress irrelevant information (Bregman 1990); this type of processing appears impaired in people with DD (Sutter et al. 2000). More specifically, several studies have reported impaired ability to disentangle speech from background noise in individuals with DD (e.g. Boets et al. 2007; Calcus et al. 2018; Dole et al. 2012; Nittrouer et al. 2018; van Hirtum et al. 2019; Ziegler et al. 2009). However, there is also evidence to the contrary (Messaoud-Galusi et al. 2011) as well as evidence pointing to variable performance in DD and comorbidities that may account for these speech-in-noise impairments (Mari et al. 2022). Deficits in speech perception in noise in DD are also moderated by performance measures, i.e., accuracy versus threshold (Li et al. 2023). In light of this mixed picture, we looked at whether background noise has an adverse effect on sentence repetition performance in participants with DD.

Given the comorbidity of DD and congenital amusia—the latter typically being associated with lifelong impairments in perception and production of pitch (Peretz 2016)—reported in recent studies (Couvignou et al. 2019; Couvignou and Kolinsky 2021), we screened all our participants for amusia to establish whether the documented rate can be replicated in a new adult sample with DD.

The primary objective of the current study was to determine whether sung speech would confer an advantage in performance among neurotypicals and, in particular, participants with DD. Secondary objectives included understanding whether embedding babble noise in the stimuli would impair sentence repetition, and whether sentence type—semantically anomalous, story-like, and news-like sentences—would modulate performance. Based on the literature reviewed above, we expected sung speech to facilitate performance, particularly in participants with DD. We also anticipated that noise-embedded stimuli and semantically anomalous content would be associated with poorer performance.

## Methods

### Participants

Fifty-six monolingual native British English participants were recruited online on Prolific.com and remunerated for two hours of their time. Participants were included only if they were monolingual native speakers of British English and had no formal music training. Half of them (*n* = 28) had a diagnosis of dyslexia and an average age of 30.39 years (*SD* = 10.84); the remaining participants (*n* = 28) were controls with an average age of 32.61 years (*SD* = 9.83) and no known developmental conditions. Both groups were screened for amusia using the Montreal Battery of Evaluation of Amusia (Peretz et al. 2003; see Materials for details). Table 1 presents participant demographics and background measures.

**Table 1:** Demographics and performance on background tasks

Measure	Controls	Dyslexics	t	*p*
*n*	28	28		
Age	30.39 (10.84)	32.61 (9.83)	-0.80	0.427
Gender	6 M; 22 F	3 M; 25 F		
Language background	English monolingual	English monolingual	NA	NA
Reported tinnitus	1/28	2/28		
**Digit span**	**5.86 (1.38)**	**4.68 (2.23)**	**2.38**	**0.022**
Years of music training	1.28 (2.85)	0.42 (1.91)	1.32	0.19
Deary-Liewald (simple trials)	326.42 (132.57)	326.64 (115.06)	-0.03	0.97
**Deary-Liewald (choice trials)**	**469.94 (124.60)**	**485.42 (144.80)**	**-2.26**	**0.02**
*MBEA*				
Scale	25.89 (2.63)	25.61 (2.86)	0.39	0.699
**Contour**	**24.21 (2.82)**	**22.18 (3.99)**	**2.20**	**0.032**
**Interval**	**24.46 (2.41)**	**22.39 (4.09)**	**2.31**	**0.026**
**Rhythm**	**24.25 (3.40)**	**22.07 (4.48)**	**2.05**	**0.046**
Meter	23.54 (5.28)	20.43 (6.94)	1.89	0.065
**Memory**	**26.18 (2.83)**	**23.89 (4.69)**	**2.21**	**0.032**
**Pitch composite**	**74.57 (6.02)**	**70.18 (9.34)**	**2.09**	**0.042**
**Global MBEA**	**0.8 (0.07)**	**0.76 (0.11)**	**2.77**	**0.008**

*M* male, *F* female, *MBEA* Montreal Battery of Evaluation of Amusia

For continuous measures, the second and third columns from the left present the means, followed by standard deviations in parentheses. The maximum achievable score for the MBEA subtests is 30. The pitch composite score was obtained by adding the scores from the first three subtests; Global MBEA is the average percentage of correct responses on the six subtests. Statistically significant results are shown in bold (*p* < 0.05). As controls are the first group listed, negative values indicate that they were outperformed by participants with DD. It should be noted, however, that in the Deary–Liewald task, values correspond to reaction times (in milliseconds), with longer reaction times reflecting slower (and therefore poorer) performance. All participants reported normal hearing unless otherwise indicated in the tinnitus row

### Materials

One experimental task and other background tests were administered to participants using Gorilla, an online testing platform (Anwyl-Irvine et al. 2020). The materials are described below in the order they were presented to participants.

The Forward Digit Span (Wechsler Adult Intelligence Scale IV; Wechsler 2008). To complete this task, participants are required to type gradually increasing visually presented sequences with a minimum of two and a maximum of nine digits. Participants in our study were presented with two trials per digit length and a maximum of 16 trials in total. If they were unable to produce the correct sequence two consecutive times, no more trials were administered.

*The Sentence Repetition Task.* This was the main experimental task, previously used in Loutrari et al. (2024) in English and Zhao et al. (2024) in Mandarin Chinese. We used a mixed design to assess sentence repetition, with one between-subjects variable (group: controls and participants with DD) and three within-subjects variables (mode of presentation, noise level, and semantic content). To complete this task, participants are required to listen to 60 utterances (excluding six practice trials) and record themselves repeating them back by pressing a button. They are instructed to repeat back any words they hear if they cannot reproduce full sentences. Targets are primarily simple declarative sentences, with only a small number containing an embedded clause. There are two modes of presentation, namely spoken and sung, but participants are asked to always repeat sentences back in the speech modality. The utterances are ten words long on average, with three different types of semantic content: expository (news-like), narrative (story-like), or semantically anomalous (meaningless but with preserved syntactic structure). As detailed in Loutrari et al. (2024), spoken stimuli in the task have a mean duration of 2.61 s, while sung stimuli have a mean duration of 5.53 s. The mean F_0_ in spoken and sung stimuli is 242.6 Hz and 301.50 Hz respectively. The average minimum and maximum frequencies in spoken stimuli are 150.14 Hz and 316.82 Hz respectively, and the corresponding values in sung stimuli are 184.18 and 385.73 Hz. The mean tempo in bpm is 113 for sung stimuli (this measure did not apply to the spoken targets). All stimuli have been adjusted to a root-mean-square (RMS) amplitude of 0.1 Pa (equivalent to 74 dB). Two noise levels are included; targets are either presented in quiet or mixed with babble noise with a signal-to-noise ratio of 5 dB. For more details on the acoustic features of the stimuli and the babble noise as well as the semantic content of the targets and the recording procedure, the reader can refer to Loutrari et al. (2024).

A Latin square design with four lists resulted in different presentation orders for sung and spoken targets as well as noisy and quiet conditions. Each list contained the same sentences representing all three semantic conditions, but the combination of variables varied so that a given sentence appeared with or without noise and in sung or spoken form across lists.

Four raters transcribed and scored the obtained responses. Participants were penalised for derivational errors (e.g. saying ‘explain’ instead of ‘explanation’) but not for repeating words in the wrong order, adding words, or making inflectional errors (e.g. saying ‘explain’ instead of ‘explains’) as in Loutrari et al. (2024). In line with previous studies (Liu et al. 2015; Loutrari et al. 2024; Madsen et al. 2017; Zhao et al. 2024), we computed the percentage of correct words transformed into RAU (Rationalised Arcsine Units) (Studebaker 1985).

*The Montreal Battery of Evaluation of Amusia (MBEA)* (Peretz et al. 2003). This diagnostic battery comprises six subtests assessing different facets of music perception. The first four subtests consist of pairs of melodies with a reference and a comparison melody requiring judgements related to pitch (scale, contour and interval subtests) and rhythm (rhythm subtest); they contain 31 trials each including a catch trial. To complete the fifth subtest (meter), participants are tasked with distinguishing marches from waltzes. The last subtest (memory) is an incidental memory task whereby participants are instructed to distinguish between melodies they heard during the task and completely novel melodies. The last two subtests include 30 trials each. Our participants were given two to four practice trials across subtests. In addition to the scores from each subtest, we extracted a composite pitch score (the sum of the scale, contour, and interval subtests) and a global score (the average percentage of correct responses for the whole battery).

*The Deary-Liewald task* (Deary et al. 2011). This task requires participants to press the spacebar upon seeing a diagonal cross on their screens to complete the first part of the task. In the second part, they are asked to press one of four response keys depending on the position of the cross. Our participants were given five practice trials and 40 experimental trials. Two separate scores were extracted based on the total number of correct responses. The scores reported in Table 1 correspond to reaction times in milliseconds, with longer reaction times indicating worse performance.

### Procedure

Participants started by filling out a questionnaire on background information, covering age, gender, music background, and medical history (see Table 1). They were subsequently presented with a description of the study and a consent form, which they signed before attempting the tasks. Participants used their personal devices and headphones to complete the tasks. Testing lasted 90 min on average excluding breaks (participants were encouraged to take breaks as they saw fit), and a debrief was shared with them upon completion of the tasks.

## Results

**Fig. 1:**
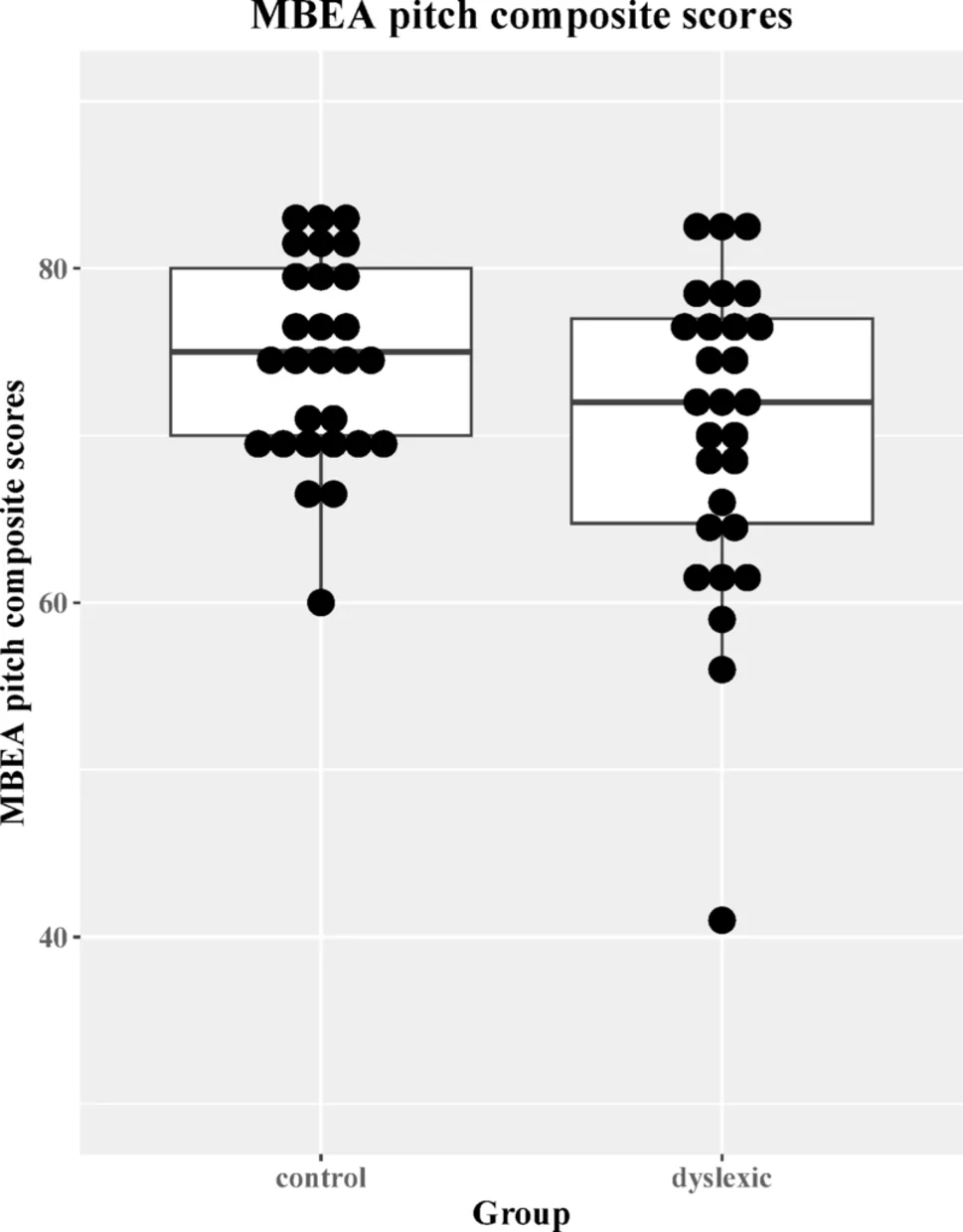
Pitch composite score (sum of the pitch-based MBEA subtests) achieved by controls and participants with DD. The boxplots show the quartiles and the median (thick horizonal line); filled circles represent individual participants. See Table 1 for more descriptive statistics on the pitch composite score

This section reports on the results from a linear mixed-effects model with four fixed effects, namely group, noise level, semantic content, and mode of presentation, and two random effects: participants and items. Satterthwaite approximations were used to estimate degrees of freedom. In addition to the main experimental portion of the study, we screened participants for amusia using the MBEA (see Table 1 in Methods for a comparison between controls and participants with DD). Participants who achieved a pitch composite score (sum of the first three subtests) of 65 or less (Liu et al. 2010) were diagnosed as amusic. One control and eight participants with DD (approximately 29% of the DD sample) met this criterion. Figure 1 compares the performance of the two groups on the MBEA based on this aggregate score.

As some of the participants in the sample had amusia, we considered possible effects of amusia on performance, but it was not found to significantly affect results, *F*(1, 55.1) = 0.38, *p* = 0.54, η_*p*_^*2*^ = 0.006. Given that the experiment relied on verbal short-term memory – and participants with DD had a lower digit span – we also input digit span in the model, but again, it was not found to significantly affect sentence repetition performance, *F*(1, 163.8) = 0.23, *p* = 0.63, η_*p*_^*2*^ = 0.001. Similarly, a model including the pitch composite score as a predictor showed that it did not have an effect on sentence repetition (see Table 2). The analysis below reports on a simpler model that does not include digit span, amusia, or the pitch composite score as predictors.

**Table 2:** Main effects and interactions from the linear mixed-effects model

	NumDF	DenDF	F	*p*	η_*p*_^2^
**Group**	**1**	**54.00**	**10.09**	**0.002**	**0.157**
Presentation	1	3246.18	1.98	0.159	< 0.001
**Noise level**	**1**	**3258.90**	**727.87**	**< 0.001**	**0.183**
Semantic content	2	982.50	0.72	0.485	0.001
Group × presentation	1	3258.90	0.18	0.671	< 0.001
**Group × noise level**	**1**	**3246.18**	**51.60**	**< 0.001**	**0.016**
**Presentation × noise level**	**1**	**3256.23**	**21.15**	**< 0.001**	**0.006**
Group × semantic content	2	3259.32	2.66	0.070	0.002
Presentation × semantic content	2	3249.37	1.01	0.364	< 0.001
**Noise level × semantic content**	**2**	**3262.14**	**9.67**	**< 0.001**	**0.006**
Group × presentation × noise level	1	3231.73	0.004	0.945	< 0.001
Group × presentation × semantic content	2	3255.75	0.25	0.777	< 0.001
Group × noise level × semantic content	2	3243.15	0.09	0.911	< 0.001
**Presentation × noise level × semantic content**	**2**	**3266.65**	**4.52**	**0.011**	**0.003**
Group × presentation × noise level × semantic content	2	3224.90	0.09	0.912	< 0.001
Pitch composite *	1	52.1	0.17	0.682	0.003
Pitch composite × group *	1	51.9	1.16	0.285	0.02

*NumDF,* numerator degree of freedom; *DenDF,* denominator degree of freedom; Presentation, mode of presentation

Significant effects and interactions are marked in bold (*p* < 0.05). Rows marked with an asterisk were obtained from a separate model including the pitch composite score as a predictor

The main analysis revealed a significant effect of group, *F*(1, 54) = 10.09, *p* = 0.002, η_*p*_^*2*^ = 0.157, with controls significantly outperforming participants with DD. There was also a significant effect of noise, *F*(1, 3259) = 727.87, *p* < 0.001, η_*p*_^*2*^ = 0.183, with sentences presented in quiet being more accurately recalled, but not a significant main effect of semantic content, *F*(2, 982) = 0.72, *p* = 0.485, η_*p*_^*2*^ = 0.001. A significant group × noise interaction was also observed, *F*(1, 3246) = 51.60, *p* < 0.001, η_*p*_^*2*^ = 0.016, albeit with a small effect size. Post hoc pairwise comparisons with FDR corrections showed that participants with DD performed significantly more poorly in quiet, *t*(65.3) = 5.19, *p <* 0.001, *d =* 1.285, but on par with controls in noise, *t*(65.3) = 0.87, *p =* 0.388, *d =* 0.215. There was no significant effect of mode of presentation, *F*(1, 3246) = 1.98, *p* = 0.159, η_*p*_^*2*^ < 0.001, nor a significant interaction between group and mode of presentation, F(1, 3259) = 0.18, *p* = 0.671, η_*p*_^*2*^ < 0.001.

Although the effect sizes for the group and noise level main effects were large, the four significant interactions that we report (group × noise level, presentation × noise level, noise level × semantic content, and presentation × noise level × semantic content) are small (see Discussion for elaboration).

**Fig. 2:**
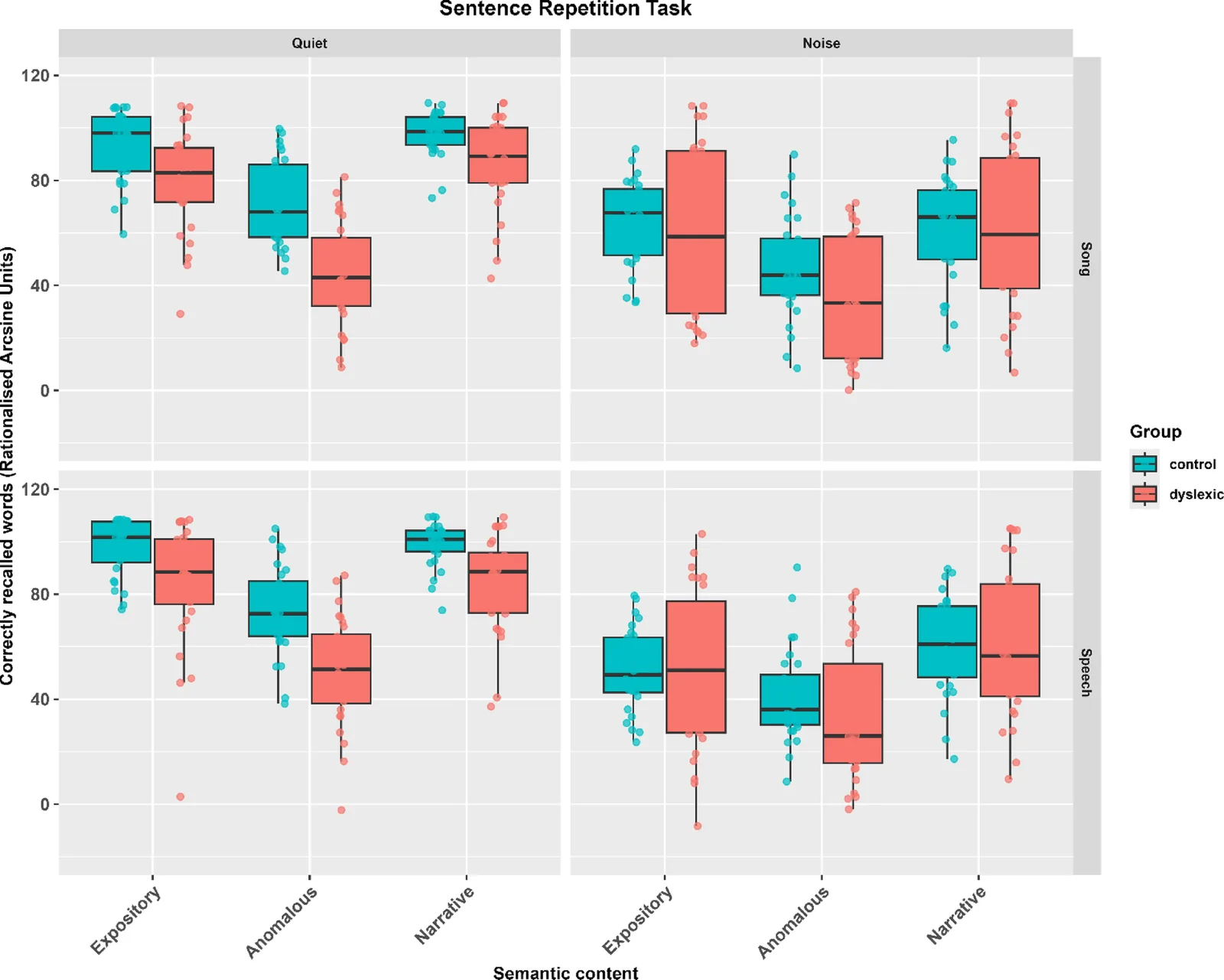
Boxplots presenting participants’ performance (in Rationalised Arcsine Units) across all conditions. The whisker boxes display the quartiles and the median. In spoken trials, controls (*n* = 28), averaged 51.6 RAU (*SD* = 34.7) in the noise condition, and participants with DD (*n* = 28) had an average RAU of 52.1 (*SD* = 40.9). In the quiet condition, controls had an average RAU of 90.5 (*SD* = 24.8), and participants with DD had an average RAU of 71.7 (*SD* = 35.6). In the sung trials, controls (*n* = 28), averaged 57.10 RAU (*SD* = 33.10) in the noise condition, and participants with DD (*n* = 28) had an average RAU of 53.2 (*SD* = 39.40). In the quiet condition, controls had an average RAU of 87.41 (*SD* = 28.70), and participants with DD had an average RAU value of 69.90 (*SD* = 38.30)

Results are also shown in Fig. 2.

While the finding that participants with DD performed worse in quiet but not in noise is counterintuitive, we did not probe further into combinations of variables, as all other interactions involving the group variable came out non-significant. Table 2 presents the full set of main effects and interactions including non-significant results and significant results that are not considered further here due to negligible effect sizes.

We computed a series of correlations using Holm’s correction for multiple comparisons to gauge possible relationships between sentence repetition performance and MBEA global scores. There was no significant correlation between spoken sentence repetition and MBEA global scores for participants as a whole (*r* = 0.33, *p* = 0.070), or separately for controls (*r* = 0.30, *p* = 0.348) and participants with DD (*r* = 0.19, *p* = 0.376). A significant correlation was observed for repetition of sung sentences and MBEA global scores for participants as a whole (*r* = 0.40, *p* = 0.012). Although the magnitude of the correlation coefficient was even stronger for the control group in isolation, the corrected *p* value did not come out significant (*r* = 0.44, *p* = 0.072). A smaller non-significant correlation was seen for the group with DD (*r* = 0.26, *p* = 0.376).

## Discussion

Overall, babble noise compromised performance across participants, but the mode of presentation (i.e. stimuli being presented in a spoken versus a sung form) and the semantic content of the utterances were not associated with significant differences in participants’ scores. Participants with DD performed more poorly relative to neurotypical controls when repeating back spoken and sung targets; they achieved a lower score than controls in quiet but not in noise. Sung targets did not seem to have a facilitatory effect on the performance of listeners with DD. In addition to the main experimental task, we found that participants with DD were significantly outperformed by controls on all but two MBEA measures. Further, amusia screening showed that approximately 29% of the DD sample met the criteria for amusia.

Our findings are in keeping with previous literature showing impaired sentence repetition in DD (Dosi and Koutsipetsidou 2019; Lukic et al. 2025; Moll et al. 2015; Plaza et al. 2002). However, although sentence repetition in participants with DD was found to be impaired in quiet, this was not the case in noise. This is in line with some previous work (Messaoud-Galusi et al. 2011) but contrasts with various other studies pointing to impaired speech-in-noise perception in DD (Boets et al. 2007; Calcus et al. 2018; Dole et al. 2012; Li et al. 2023; Nittrouer et al. 2018; van Hirtum et al. 2019; Ziegler et al. 2009). Speech perception and speech-in-noise perception are rather broad terms, and hence a direct comparison with previous findings is not straightforward. More specifically, in some of these studies participants had to repeat words rather than sentences. There is also considerable variation in masking conditions in the literature. Furthermore, heterogeneity in the disorder complicates comparisons across studies; in a recent study, children with DD were found to have typical speech perception in quiet and in noise unless they had co-morbid speech sound disorder (Mari et al. 2022). Yet a recent meta-analytic review suggested that participants with DD not only show deficits in reading comprehension but also in listening comprehension (Georgiou et al. 2022). Thus, our finding of impaired sentence repetition in quiet in DD might be linked to their impaired oral language skills (Snowling and Hulme 2021).

Furthermore, several other possibilities may explain the seemingly paradoxical pattern of worse repetition in quiet but comparable performance in noise in DD relative to controls. First, attentional modulation can improve speech encoding under challenging listening conditions (Price and Bidelman 2021), and individual differences in such attentional modulation predict speech-in-noise performance (Saiz-Alía et al. 2019). This could allow some participants with DD to compensate in the noisy condition. Second, arousal effects (Han et al. 2013) mean that adding low-arousal speech noise may improve or normalise performance for individuals with DD who are under-aroused in quiet. Third, stochastic resonance/noise-benefit mechanisms can, under specific intensities, enhance detection or discrimination of weak auditory signals so that a low level of external noise actually improves speech encoding and recognition (Herrmann 2024; Matthews et al. 2024). Importantly, these mechanisms are not mutually exclusive; a mild noise level could both increase arousal and enable stochastic resonance while also eliciting greater attentional engagement in listeners who find quiet conditions less stimulating. However, these explanations remain speculative and await future investigation.

The literature on the effects of music- and auditory-based interventions on reading difficulties in children has produced mixed results (Cancer and Antonietti 2022). In our study, sung utterances did not seem to enhance the performance of participants with DD, as suggested by the absence of a significant interaction between group and mode of presentation. Put differently, presenting utterances in a sung form was not found to compensate for compromised sentence repetition ability associated with DD (Dosi and Koutsipetsidou 2019; Lukic et al. 2025; Moll et al. 2015; Plaza et al. 2002). The performance of the whole sample was statistically comparable across the spoken and sung conditions. Previous work in neurotypicals has shown better recall for sung relative to spoken lyrics due to differences in presentation rate (Kilgour et al. 2000). However, it is of note that participants in the study by Kilgour and colleagues were exposed four times to song verses as opposed to sentences presented once in 60 separate trials. Moreover, their study employed poetic lyrics with rhyming patterns, whereas we used news-like, story-like, and semantically anomalous sentences. Finally, the sung stimuli used in this study were produced by an amateur singer, not a trained vocalist as in Kilgour et al. (2000). The present results, however, also diverge from Loutrari et al. (2024) whereby the same sung utterances were associated with better recall in noise (but not in quiet) in another neurotypical sample. It is worth mentioning that Kilgour et al. (2000) did not report effect sizes and the effect size of the comparison in Loutrari et al. (2024) is small. Our results are more in line with those of Racette and Peretz (2007), although their design and testing procedure were considerably different to ours. The current study showed that naturalistic singing was not associated with benefits for sentence repetition, but this does not constitute evidence that lower presentation rate per se does not confer benefits. Future research with multiple rate conditions and potentially only sung speech (to avoid ecologically non-valid linguistic stimuli) can clarify whether different rates of singing yield any measurable effects.

Results did not reveal a significant main effect of semantic content, which is somewhat surprising given previous evidence that semantically meaningful content facilitates sentence repetition (Polišenská et al. 2015, 2021). This may be due to the content being challenging across categories. Although in Loutrari et al. (2024) there were some significant interactions between semantic content and other variables, the current findings replicate the absence of a main effect of semantic content.

Turning again to participants with DD, individual differences may have a bearing on the absence of a compensatory singing effect (Christiner et al. 2022); rapid auditory processing ability in DD may be modulated by additional variables including dyslexia subtype (surface versus phonological), age, and a previous diagnosis of language impairment (Lorusso et al. 2014; see also limitations below). At the same time, auditory processing difficulties may constitute only one contributing factor in DD (Leppänen et al. 2010) and may not be equally captured across different testing conditions (Witton et al. 2020).

The finding that approximately 29% of our DD sample also met the criteria for amusia concurs with previous evidence in adults (Couvignou et al. 2019). The comorbidity of the two disorders and their possibly shared underpinnings have been the subject of recent investigations (Couvignou et al. 2019, 2023, 2024; Couvignou and Kolinsky 2021). Earlier work had suggested that, although congenital amusia is typically seen as a disorder primarily affecting music perception, amusia and dyslexia may both relate to an awareness deficit (Peretz 2016). Recent experimental work has alternatively suggested that the comorbidity may stem from shared impairments in auditory serial-order short-term memory (Couvignou et al. 2023). While the underlying connection between the two disorders is well beyond the scope of the present study, our findings corroborate the documented extent of their overlap. However, the presence of amusia had no impact on sentence repetition accuracy, replicating the results of Loutrari et al. (2024); dyslexia was the only group characteristic found to affect performance. The presence of amusia in samples with DD does not necessarily indicate shared underlying mechanisms. Rather, it shows that DD is more heterogeneous than previously thought, making it more challenging to characterise and study. However, the evidence does not allow for definitive conclusions in relation to amusia, as task complexity and participants’ language background—among other potential variables—may also exert an effect. It is of note that in Liu et al. (2015), Mandarin-speaking participants with amusia were outperformed by controls in a similar speech intelligibility task both in quiet and in noise.

## Limitations and conclusions

The signal-to-noise ratio employed in the study was not particularly demanding and does not reflect the full range of real-world noisy listening conditions. Further, unlike tasks requiring repetition of isolated words or pseudowords, our task may have allowed participants to rely partially on syntactic structure to determine the appropriate response. Information regarding participants’ history of speech therapy and its duration was not available, and Speech Sound Disorder (which may be of relevance to the observed outcomes) was not screened for, adding to the study’s limitations.

It should also be noted that the rate of presentation was not isolated from other possibly conflating variables in the sentence repetition task; pitch contours, for example, were not matched across modes of presentation (as such, manipulation would render either the spoken or the sung stimuli less ecologically valid). An alternative design could compare spoken and sung sentences presented at different rates to determine whether performance differences (if any) are attributable to the rate or mode of presentation. Even so, ruling out the effects of other differences between spoken and sung material while preserving the ecological validity of the stimuli may prove challenging.

In conclusion, the study found participants with DD to be less adept at sentence repetition relative to neurotypicals. At this juncture, it is not clear why the difference in performance was not amplified by noise. Given the multifactorial origins and manifestations of DD (Hari and Renvall 2001; Snowling and Hulme 2021), further research is warranted to determine the exact experimental conditions that may account for variation in speech perception and sentence repetition across the literature. An alternative approach may be also needed to better understand heterogeneity in the disorder, identifying causal mechanisms and characterising differences in perception, recall, and repetition.

## Figures and Tables

**Fig. 3:**
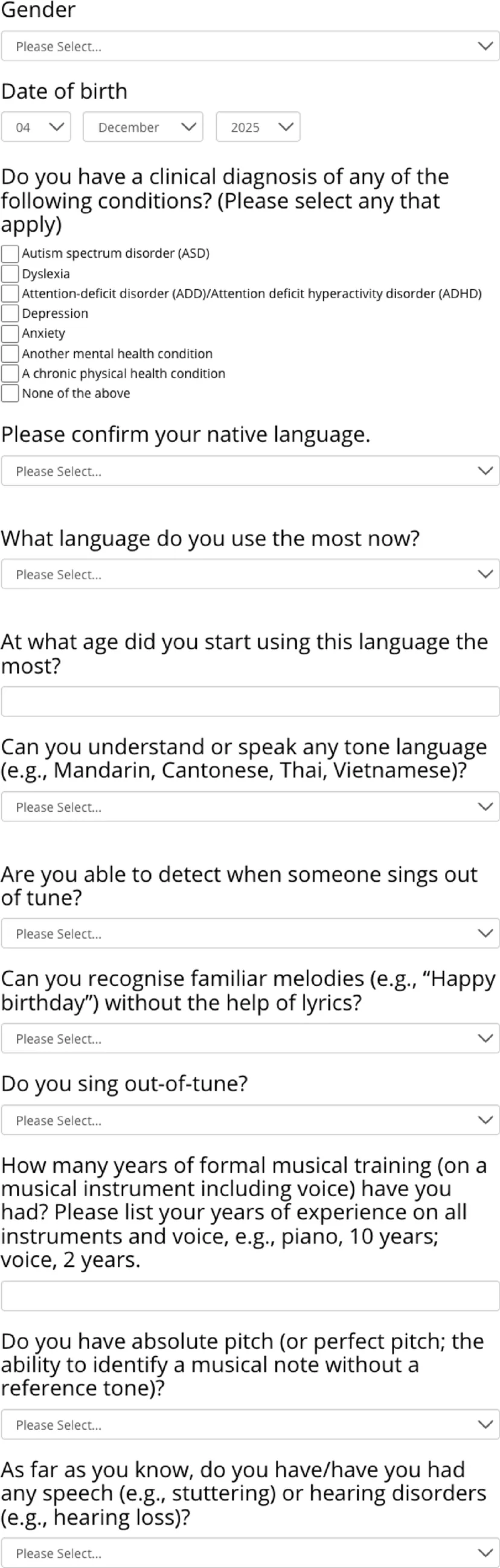
Background information questionnaire

## Appendix

See Fig. 3
